# Risk of adverse birth outcomes after adolescent and young adult cancer

**DOI:** 10.1093/jncics/pkad106

**Published:** 2023-12-21

**Authors:** Chelsea Anderson, Christopher D Baggett, Stephanie M Engel, Darios Getahun, Nancy T Cannizzaro, Sara Mitra, Clare Meernik, Lisa M Moy, Cecile A Laurent, Xi Zhou, Lanfang Xu, Marilyn L Kwan, William A Wood, Barbara Luke, Chun R Chao, Lawrence H Kushi, Hazel B Nichols

**Affiliations:** Center for Gastrointestinal Biology and Disease, University of North Carolina, Chapel Hill, NC, USA; Department of Epidemiology, University of North Carolina, Chapel Hill, NC, USA; Lineberger Comprehensive Cancer Center, University of North Carolina, Chapel Hill, NC, USA; Department of Epidemiology, University of North Carolina, Chapel Hill, NC, USA; Department of Research and Evaluation, Kaiser Permanente Southern California, Pasadena, CA, USA; Department of Research and Evaluation, Kaiser Permanente Southern California, Pasadena, CA, USA; Lineberger Comprehensive Cancer Center, University of North Carolina, Chapel Hill, NC, USA; Department of Population Health Sciences, Duke University School of Medicine, Durham, NC, USA; Division of Research, Kaiser Permanente Northern California, Oakland, CA, USA; Division of Research, Kaiser Permanente Northern California, Oakland, CA, USA; Lineberger Comprehensive Cancer Center, University of North Carolina, Chapel Hill, NC, USA; MedHealth Statistical Consulting Inc, Solon, OH, USA; Division of Research, Kaiser Permanente Northern California, Oakland, CA, USA; Lineberger Comprehensive Cancer Center, University of North Carolina, Chapel Hill, NC, USA; Division of Hematology, Department of Medicine, University of North Carolina, Chapel Hill, NC, USA; Department of Obstetrics, Gynecology, and Reproductive Biology, College of Human Medicine, Michigan State University, East Lansing, MI, USA; Department of Research and Evaluation, Kaiser Permanente Southern California, Pasadena, CA, USA; Division of Research, Kaiser Permanente Northern California, Oakland, CA, USA; Department of Epidemiology, University of North Carolina, Chapel Hill, NC, USA; Lineberger Comprehensive Cancer Center, University of North Carolina, Chapel Hill, NC, USA

## Abstract

**Background:**

Many women diagnosed with cancer as adolescents and young adults (AYAs, age 15-39 years) want biological children after cancer but lack information on the potential impact of their cancer history on future reproductive outcomes. We investigated the risk of adverse birth outcomes among AYA cancer survivors.

**Methods:**

We identified insured women diagnosed with AYA breast cancer, thyroid cancer, gynecologic cancers, lymphoma, or melanoma from 2003 to 2016 in the state of North Carolina or the Kaiser Permanente health care systems in northern and southern California. Post-diagnosis births to cancer survivors were each matched with up to 5 births to women without cancer. Risk ratios for preterm birth (<37 completed weeks), very preterm birth (<34 completed weeks), low birth weight (<2500 g), and small for gestational age (SGA, <10th percentile of weight for gestational age) were estimated using modified Poisson regression.

**Results:**

Analyses included 1648 births to 1268 AYA cancer survivors and 7879 births to 6066 women without cancer. Overall, risk of preterm birth, very preterm birth, low birth weight, and SGA did not significantly differ between births to women with and without cancer. However, births to women with gynecologic cancers had a significantly increased risk of low birth weight (risk ratio = 1.82; 95% confidence interval: 1.03 to 3.21) and suggested increased risk of preterm birth (risk ratio = 1.59; 95% confidence interval: 0.99 to 2.54). Chemotherapy exposure was not associated with increased risk of adverse birth outcomes.

**Conclusions:**

Women with gynecologic cancers, but not other cancers, had an increased risk of adverse birth outcomes compared to women without cancer.

Cancers diagnosed among adolescents and young adults (AYAs, age 15-39 years) exceed 87 000 each year in the United States (1) and coincide with a critical life stage for decisions around childbearing and parenthood. With high 5-year survival (>85%) for all cancers combined in this age group (1), many AYA women will live long enough to have biological children after cancer, but they may have concerns about the potential impact of cancer treatment on their future reproductive outcomes. These concerns may relate not only to subsequent fertility but also to the risk of adverse birth outcomes for those who do become pregnant.

Several recent studies have investigated births to cancer survivors, with most finding a small to moderate increase in the risk of preterm birth and low birth weight (LBW), but no difference in risk of small for gestational age (SGA) births, compared with births to women without a history of cancer (2-16). However, these studies have often included births to childhood cancer survivors (diagnosed before age 15 years) (6,7,9,10,12-14), who may differ substantially from AYA cancer survivors in their distribution of cancer types, cancer treatment-related exposures, and interval between cancer diagnosis or treatment and subsequent pregnancy. Most studies focused on survivors diagnosed with cancer as AYAs have come from Europe (3-5) and therefore may not reflect patterns of cancer or reproductive health care for US patients or rates of adverse birth outcomes in the US general population. Many prior studies have also lacked cancer treatment information (3,5-7,9,10,13), which is critical for understanding whether exposure to certain therapies contributes to the excess risk of preterm birth or other adverse outcomes among survivors. Additional evidence is needed to inform reproductive counseling for US cancer patients diagnosed and treated as AYAs.

In this study, we examined the risk of adverse birth outcomes among insured AYA cancer survivors in North Carolina and California compared with a matched sample of women without cancer. Adverse birth outcomes included preterm birth, LBW, SGA, low Apgar scores, and birth defects. We focused on the most commonly diagnosed cancer types in AYA women in the United States and considered how risk differed according to maternal and cancer-related characteristics.

## Methods

### Study design and population

These analyses use data from the AYA Horizon study, a cohort study established to investigate pregnancy outcomes among women diagnosed with cancer as AYAs (17). We identified women diagnosed with invasive or in situ breast cancer or with invasive thyroid cancer, melanoma, lymphoma, and gynecologic (ovarian, cervical, or uterine cancers) cancers, at age 15-39 years in North Carolina (2003-2015) or the Kaiser Permanente Northern California (KPNC) and Southern California (KPSC) health care systems (2004-2016). Included cancer types represent the most common cancer types in this age group [>70% of all cancers among female AYAs (18)]. For women in KPNC or KPSC, cancer diagnosis and treatment information came from the health system cancer registries. Information on maternal characteristics and birth outcomes was obtained from health system birth registries, electronic health records (EHRs), and membership files. Birth defects were defined based on International Classification of Diseases (ICD-9 and ICD-10) codes. For women in North Carolina, state cancer registry records were linked to statewide birth certificate files to ascertain information on cancer diagnosis, birth outcomes, and maternal characteristics. These data were also linked with the North Carolina Birth Defects registry, which conducts active surveillance to identify birth defects diagnosed within the first year of life. Cancer treatment information came from linkage of cancer registry records and public and private payer administrative insurance claims from the University of North Carolina (UNC) Cancer Information Population Health Resource (CIPHR) (19). The CIPHR linkage covers 80% of cancers diagnosed in North Carolina through 2015. Data from all 3 sites were also linked to the Society for Assisted Reproductive Technology Clinic Outcomes Reporting System (SART CORS) database, which covers greater than 90% of assisted reproductive technology (ART) procedures in the United States since 2004 (20). The study was approved by Institutional Review Boards at UNC, KPNC, and KPSC with a waiver of informed consent.

For inclusion in these analyses, we required women to be enrolled in the KPNC or KPSC health care systems or private insurance in North Carolina both at the time of cancer diagnosis and continuously for 6 months before diagnosis (allowing 90-day gaps). Women enrolled in Medicaid in North Carolina were required to be enrolled within 1 month of diagnosis; they were not required to have continuous enrollment before diagnosis because a cancer diagnosis may be a qualifying event for Medicaid enrollment. For women in North Carolina, we also required 6 months of continuous enrollment in private insurance or Medicaid after diagnosis (no gaps) for ascertainment of cancer treatment information.

Among eligible women, we identified 2039 births after an AYA cancer diagnosis. These were matched to up to 5 births to women without a history of cancer on maternal age, year of delivery, study site, and for KPNC and KPSC, health system enrollment year (N = 10 190). We excluded births when the cancer diagnosis occurred during pregnancy (N = 274) and their matched noncancer births (N = 1372); non-singleton births (N = 94 cancer, 612 noncancer); and nonviable births (<500 g or <20 weeks gestation) and births missing data on birthweight, gestational age, and/or infant sex (N = 23 cancer, 327 noncancer).

### Outcomes

Adverse birth outcomes were identified using birth certificate data in North Carolina and health system records in KPNC and KPSC. Outcomes of interest included preterm birth (<37 completed weeks of gestation), very preterm birth (<34 completed weeks of gestation), LBW (<2500 g), SGA (<10th percentile of weight for gestational age) (21), low 5-minute Apgar score (<7), and any birth defect (≥1 vs none).

### Covariates

Maternal and birth characteristics, including year of birth, maternal age, race and ethnicity, marital status, gestational diabetes, pre-pregnancy body mass index (BMI), and smoking during pregnancy were abstracted from state birth certificates in North Carolina. For KPNC and KPSC, these data were obtained from EHRs and other clinical and administrative databases. For all study sites, information on cryopreservation and transfer of embryos or oocytes came from SART CORS. Among AYA cancer survivors, we defined fertility preservation as embryo or oocyte cryopreservation (without transfer) within 90 days of a cancer diagnosis.

Cancer characteristics from the state cancer registry in North Carolina and health system cancer registries for KPNC and KPSC included cancer type, cancer stage, and age at diagnosis. Cancer types were defined using AYA-specific recodes of the International Classification of Diseases for Oncology, Third Edition (ICD-O-3) topography and histology codes (22). Information on specific chemotherapy agents, including alkylating agents and anthracyclines, received within 12 months of cancer diagnosis came from EHR data at KPNC and KPSC and insurance claims in North Carolina. For women with gynecologic cancers and lymphoma, radiation receipt and field were abstracted from EHR data or medical charts for KPNC and KPSC and insurance claims in North Carolina. Procedure codes from the ICD-9/10, Healthcare Common Procedure Coding System (HCPCS), and Common Procedural Terminology (CPT) were used to identify chemotherapy and radiation oncology administrations. Lists of prescription drug HCPCS and National Drug Codes (NDC) were used to determine specific chemotherapy agents used. These lists were developed from collaborator input, lists from the Cancer Research Network (23), and a Surveillance, Epidemiology, and End Results-Medicare report (24).

### Statistical analyses

We estimated risk ratios (RRs) for adverse birth outcomes using modified Poisson regression models with generalized estimating equation (GEE) methods to account for multiple births occurring to the same mother (25). Multivariable models were adjusted for year of birth, maternal age, race and ethnicity, marital status, and maternal smoking during pregnancy. Stratified analyses of preterm birth, LBW, SGA, and birth defects were performed according to maternal characteristics and cancer characteristics. We did not perform stratified analyses for very preterm birth and low Apgar score due to the small number of events for these outcomes. Stratified analyses according to cancer treatment were performed only for chemotherapy; sample sizes were too small for multivariable analyses stratified by other treatment exposures. For all stratified analyses, we did not estimate a RR if the number of adverse birth outcomes in the cancer group in that stratum was less than 10. All statistical tests were 2-sided, and *P* values less than 0.05 were considered statistically significant.

## Results

Analyses included 1648 births to 1268 women with AYA cancer and 7879 births to 6066 women without cancer. Maternal characteristics at first post-diagnosis birth in AYA cancer survivors and women without cancer are shown in Table 1. Distributions of birth year and maternal age were similar between the 2 groups due to matching. The median maternal age was 33 years (interquartile range [IQR]: 29, 36) in both groups. AYA cancer survivors were more likely to be non-Hispanic White (55%) than women without a history of cancer (42%). Distributions of marital status, gestational diabetes, pre-pregnancy BMI, and tobacco use during pregnancy were similar between groups. Few women in either the cancer (2%) or noncancer (2%) groups used ART within 12 months of their first included birth. Characteristics stratified by study site are shown in Supplementary Table 1 (available online).

**Table 1.** Maternal characteristics at first post-diagnosis births among births to AYA cancer survivors and matched births to women without cancer

	Cancer (N = 1268)	No cancer (N = 6066)
	N (%)	N (%)
**Study site**		
North Carolina	357 (28)	1718 (28)
KPSC	397 (31)	1937 (32)
KPNC	514 (41)	2411 (40)
**Maternal age at birth (years)**		
16-24	96 (8)	455 (8)
25-29	235 (19)	1123 (19)
30-34	455 (36)	2184 (36)
35-39	367 (29)	1758 (29)
40-45	115 (9)	546 (9)
Median (IQR)	33 (29, 36)	33 (29, 36)
**Year of birth**		
2003-2006	59 (5)	275 (5)
2007-2010	343 (27)	1642 (27)
2011-2013	330 (26)	1585 (26)
2014-2019	536 (42)	2564 (42)
**Maternal race and ethnicity**		
Non-Hispanic White	698 (55)	2533 (42)
Non-Hispanic Black	107 (8)	641 (11)
Hispanic	275 (22)	1810 (30)
Other	188 (15)	1082 (18)
**Marital status at birth**		
Not married	509 (40)	2386 (39)
Married	759 (60)	3680 (61)
**Tobacco use during pregnancy** ^a^		
No	1163 (96)	5518 (96)
Yes	48 (4)	251 (4)
Unknown/missing	57	297
**Pre-pregnancy body mass index** ^b^		
Underweight (<18.5 kg/m^2^)	18 (2)	92 (2)
Normal (18.5-24.9 kg/m^2^)	426 (42)	1882 (42)
Overweight (25.0-29.9 kg/m^2^)	276 (28)	1273 (28)
Obese (≥30.0 kg/m^2^)	283 (28)	1243 (28)
Unknown/missing	265	1576
**Gestational diabetes ^b^**		
No	985 (89)	4702 (89)
Yes	122 (11)	590 (11)
Unknown/missing	161	774
**Oocyte or embryo cryopreservation (without transfer) within 90 days of cancer diagnosis**		
No	1252 (99)	N/A
Yes	16 (1)	N/A
**Use of assisted reproductive technologies within 12 months of birth**		
No	1243 (98)	5956 (98)
Yes	25 (2)	110 (2)

a Unavailable for births in 2010 in North Carolina. IQR = interquartile range; KPSC = Kaiser Permanente Southern California; KPNC = Kaiser Permanente Northern California.

b Unavailable for births before 2011 in North Carolina.

Among AYA cancer survivors with a post-diagnosis birth (N = 1268), the most common cancer types were thyroid (35%), melanoma (23%), and breast (19%), followed by lymphomas (16%) and gynecologic cancers (7%) (Table 2). For cancer types other than lymphoma, most women were diagnosed at localized stage. The median age at cancer diagnosis ranged from 27 years for lymphomas to 33 years for breast cancer. Receipt of chemotherapy was common among women with lymphomas (86%) and breast cancer (61%). Few women with lymphomas or gynecologic cancers received pelvic radiation (<5%), and few women with lymphomas received total body irradiation (<5%) (data not shown).

**Table 2.** Cancer characteristics by cancer type among AYA cancer survivors with a post-diagnosis birth (N = 1268)

	Breast (N = 236)	Thyroid (N = 449)	Melanoma (N = 289)	**Lymphomas (N = 207)** ^a^	**Gynecologic (N = 87)** ^b^
	N (%)	N (%)	N (%)	N (%)	N (%)
**Cancer stage**					
In situ	34 (14)	N/A	N/A	N/A	N/A
Localized	123 (52)	256 (57)	271 (94)	45 (22)	67 (77)
Regional	68 (29)	179 (40)	9 (3)	98 (47)	14 (16)
Distant	—^c^	—	—	57 (28)	—
Unknown, unstaged, unspecified	—	—	—	7 (3)	—
**Age at cancer diagnosis (years)**					
15-24	21 (9)	107 (24)	49 (17)	78 (38)	15 (17)
25-29	42 (18)	145 (32)	95 (33)	55 (27)	30 (34)
30-34	102 (43)	130 (29)	106 (37)	52 (25)	33 (38)
35-39	71 (30)	67 (15)	39 (13)	22 (11)	9 (10)
Median (IQR)	33 (29, 35)	29 (25, 33)	30 (26, 33)	27 (23, 31)	29 (26, 32)
**Calendar year of cancer diagnosis**					
2003-2005	41 (17)	69 (15)	52 (18)	37 (18)	19 (22)
2006-2008	68 (29)	129 (29)	101 (35)	72 (35)	18 (21)
2009-2011	64 (27)	121 (27)	61 (21)	56 (27)	24 (28)
2012-2014	41 (17)	84 (19)	57 (20)	34 (16)	18 (21)
2015-2016	22 (9)	46 (10)	18 (6)	8 (4)	8 (9)
**Time between cancer diagnosis and birth (years)**					
<2	26 (11)	59 (13)	63 (21)	19 (9)	19 (22)
2-<5	149 (63)	294 (65)	158 (55)	117 (57)	53 (61)
5+	61 (26)	96 (21)	68 (24)	71 (34)	15 (17)
Median (IQR)	3 (2,5)	3 (2,4)	3 (2,4)	4 (2,5)	2 (2,4)
**Any chemotherapy**	145 (61)	—	—	179 (86)	23 (26)
Any alkylating agent chemotherapy	133 (56)	—	—	148 (71)	22 (25)
Both alkylating and anthracycline chemotherapy	98 (42)	—	—	147 (71)	—

a Includes 69 non-Hodgkin lymphoma and 138 Hodgkin lymphoma. IQR = interquartile range.

b Includes 34 cervical, 7 uterine, and 46 ovarian.

c Dashes indicate data are suppressed to comply with North Carolina requirements for reporting of small cell sizes.

In overall analyses, the risk of preterm delivery (<37 weeks) did not significantly differ between births to AYA cancer survivors and births to women without cancer (RR = 1.11; 95% CI: 0.94 to 1.32) (Table 3). Risk of very preterm birth (<34 weeks) (RR = 1.31; 95% CI: 0.93 to 1.85), LBW (RR = 1.03; 95% CI: 0.84 to 1.27), 5-minute Apgar score less than 7 (RR = 1.12; 95% CI: 0.70 to 1.81), and any birth defect (RR = 1.03; 95% CI: 0.80 to 1.33) were also not significantly different between groups. Births among AYA cancer survivors were less likely to be SGA than births to women without cancer (RR = 0.82; 95% CI: 0.69 to 0.99).

**Table 3.** Risk ratios (RR) for adverse birth outcomes comparing births among AYA cancer survivors to matched births among women without cancer

	Cancer (N = 1648)	Non-cancer (N = 7879)		
	N (%)	N (%)	Unadjusted RR (95% CI)	Adjusted RR (95% CI) ^a^
Preterm birth (<37 weeks)	149 (9)	665 (8)	1.07 (0.90 to 1.27)	1.11 (0.94 to 1.32)
Very preterm birth (<34 weeks)	41 (2)	158 (2)	1.24 (0.88 to 1.74)	1.31 (0.93 to 1.85)
Low birth weight	105 (6)	509 (6)	0.99 (0.80 to 1.21)	1.03 (0.84 to 1.27)
Small for gestational age	124 (8)	748 (9)	0.79 (0.66 to 0.95)	0.82 (0.69 to 0.99)
5-minute Apgar score <7	22 (1)	90 (1)	1.17 (0.73 to 1.85)	1.12 (0.70 to 1.81)
At least 1 birth defect	68 (4)	317 (4)	1.03 (0.79 to 1.33)	1.03 (0.80 to 1.33)

a Adjusted for year of birth, maternal age, race and ethnicity, marital status, maternal smoking during pregnancy.

In stratified analyses, RRs for adverse birth outcomes were generally similar across categories of study site, age at delivery, race and ethnicity, and gestational diabetes (Table 4). Among births to women who smoked during pregnancy, the risk of preterm birth was increased for births to AYA cancer survivors compared with births to women without a cancer history (RR = 2.26; 95% CI: 1.28 to 4.00). Births to AYA cancer survivors with an obese pre-pregnancy BMI also had an increased risk of preterm birth (RR = 1.42; 95% CI: 1.05 to 1.90) and low birth weight (RR = 1.52; 95% CI: 1.06 to 2.17). There were few clear associations in analyses stratified by cancer characteristics including cancer site, cancer stage, age at cancer diagnosis, calendar year of cancer diagnosis, time between cancer diagnosis and birth, and chemotherapy exposure (Table 5). Compared to births to women without cancer, births to women with gynecologic cancers had a borderline increased risk of preterm delivery before 37 weeks (RR = 1.59; 95% CI: 0.99 to 2.54) and a significantly increased risk of LBW (RR = 1.75; 95% CI: 1.01 to 3.02). These estimates were not meaningfully changed when births to women with an obese pre-pregnancy BMI or women who smoked during pregnancy were excluded (data not shown). Risks did not appear to be elevated for births to women with other cancer types.

**Table 4.** Risk ratios (RR) for adverse birth outcomes comparing births among AYA cancer survivors (N = 1648) to matched births among women without cancer (N = 7879), stratified by maternal characteristics

	Preterm <37 weeks	LBW	SGA	At least one birth defect
	Adjusted RR (95% CI)^a^	Adjusted RR (95% CI)^a^	Adjusted RR (95% CI) ^a^	Adjusted RR (95% CI) ^a^
**Study site**				
North Carolina	1.09 (0.79 to 1.51)	0.94 (0.65 to 1.36)	0.68 (0.48 to 0.96)	1.37 (0.83 to 2.27)
KPSC	1.00 (0.72 to 1.38)	1.21 (0.83 to 1.77)	0.97 (0.71 to 1.32)	0.97 (0.63 to 1.50)
KPNC	1.15 (0.90 to 1.48)	0.95 (0.69 to 1.30)	0.82 (0.60 to 1.10)	0.87 (0.58 to 1.32)
**Age at delivery (years)**				
16-24	1.22 (0.67 to 2.20)	NC	0.84 (0.48 to 1.47)	NC
25-29	0.89 (0.55 to 1.44)	0.92 (0.55 to 1.53)	0.75 (0.47 to 1.19)	1.19 (0.64 to 2.23)
30-34	1.25 (0.94 to 1.66)	1.22 (0.86 to 1.74)	0.83 (0.61 to 1.15)	1.36 (0.90 to 2.07)
35-39	1.10 (0.82 to 1.46)	0.97 (0.68 to 1.37)	0.84 (0.60 to 1.16)	0.81 (0.51 to 1.28)
40-45	1.00 (0.58 to 1.74)	1.15 (0.60 to 2.23)	0.90 (0.50 to 1.62)	NC
**Mother smoked during pregnancy**				
No	1.04 (0.86 to 1.25)	0.99 (0.80 to 1.24)	0.85 (0.70 to 1.04)	1.01 (0.77 to 1.32)
Yes	2.26 (1.28 to 4.00)	NC	NC	NC
**Maternal race and ethnicity**				
Non-Hispanic White	1.02 (0.78 to 1.32)	0.97 (0.70 to 1.34)	0.78 (0.59 to 1.04)	1.07 (0.75 to 1.52)
Non-Hispanic Black	1.38 (0.92 to 2.08)	1.24 (0.79 to 1.94)	0.95 (0.60 to 1.51)	NC
Hispanic	0.97 (0.67 to 1.42)	1.03 (0.65 to 1.62)	0.91 (0.61 to 1.34)	1.00 (0.58 to 1.72)
Other	1.31 (0.90 to 1.90)	1.02 (0.66 to 1.59)	0.76 (0.51 to 1.13)	NC
**Pre-pregnancy body mass index**				
Underweight (<18.5 kg/m^2^)	NC	NC	NC	NC
Normal (18.5-24.9 kg/m^2^)	0.93 (0.67 to 1.29)	0.72 (0.49 to 1.07)	0.70 (0.51 to 0.94)	1.13 (0.71 to 1.79)
Overweight (25.0-29.9 kg/m^2^)	1.07 (0.74 to 1.54)	0.89 (0.57 to 1.40)	0.83 (0.55 to 1.26)	0.90 (0.51 to 1.57)
Obese (≥30.0 kg/m^2^)	1.42 (1.05 to 1.90)	1.52 (1.06 to 2.17)	0.86 (0.55 to 1.32)	NC
**Gestational diabetes**				
No	1.11 (0.91 to 1.35)	0.99 (0.78 to 1.26)	0.86 (0.70 to 1.05)	1.00 (0.74 to 1.33)
Yes	1.05 (0.67 to 1.64)	1.11 (0.66 to 1.87)	0.67 (0.35 to 1.27)	NC

a Adjusted for year of birth, maternal age, race and ethnicity, marital status, maternal smoking during pregnancy. LBW = low birth weight; SGA = small for gestational age; NC = not calculated due to small number of outcomes; KPSC = Kaiser Permanente Southern California; KPNC = Kaiser Permanente Northern California.

**Table 5.** Risk ratios (RR) for adverse birth outcomes comparing births among AYA cancer survivors (N = 1648) to matched births among women without cancer (N = 7879), stratified by cancer characteristics

	Preterm <37 weeks	LBW	SGA	At least one birth defect
	Adjusted RR (95% CI)^a^	Adjusted RR (95% CI)^a^	Adjusted RR (95% CI) ^a^	Adjusted RR (95% CI) ^a^
**Cancer type**				
Breast	0.88 (0.57 to 1.35)	0.62 (0.34 to 1.13)	0.61 (0.37 to 0.98)	1.09 (0.60 to 2.00)
Thyroid	1.02 (0.76 to 1.37)	1.16 (0.84 to 1.61)	1.03 (0.78 to 1.35)	1.28 (0.87 to 1.90)
Melanoma	1.23 (0.84 to 1.79)	0.96 (0.59 to 1.57)	0.52 (0.30 to 0.89)	0.74 (0.39 to 1.42)
Lymphoma	1.05 (0.70 to 1.58)	0.91 (0.56 to 1.48)	0.97 (0.66 to 1.42)	0.75 (0.39 to 1.44)
Gynecologic	1.59 (0.99 to 2.54)	1.75 (1.01 to 3.02)	NC	NC
**Cancer stage**				
Localized	1.17 (0.95 to 1.45)	1.05 (0.81 to 1.37)	0.84 (0.66 to 1.06)	0.99 (0.70 to 1.39)
Regional	1.03 (0.74 to 1.43)	1.08 (0.75 to 1.58)	0.81 (0.58 to 1.14)	1.24 (0.80 to 1.92)
Distant	1.10 (0.58 to 2.09)	NC	1.08 (0.58 to 2.00)	NC
**Age at cancer diagnosis (years)**				
15-18	NC	NC	NC	NC
19-24	1.23 (0.84 to 1.82)	0.92 (0.57 to 1.49)	0.91 (0.62 to 1.34)	1.40 (0.81 to 2.41)
25-29	1.05 (0.77 to 1.44)	1.10 (0.77 to 1.59)	0.69 (0.49 to 0.99)	0.78 (0.46 to 1.32)
30-34	1.25 (0.94 to 1.67)	1.14 (0.80 to 1.61)	0.91 (0.65 to 1.28)	1.40 (0.93 to 2.11)
35-39	0.86 (0.54 to 1.37)	1.02 (0.61 to 1.71)	0.93 (0.61 to 1.44)	NC
**Calendar year of cancer diagnosis**				
2003-2005	0.79 (0.52 to 1.19)	0.86 (0.54 to 1.38)	1.00 (0.69 to 1.47)	1.02 (0.56 to 1.84)
2006-2008	1.18 (0.88 to 1.57)	0.99 (0.69 to 1.42)	0.79 (0.57 to 1.10)	0.86 (0.54 to 1.36)
2009-2011	1.31 (0.94 to 1.84)	1.00 (0.65 to 1.55)	0.68 (0.46 to 1.00)	0.98 (0.59 to 1.62)
2012-2014	1.14 (0.77 to 1.70)	1.44 (0.94 to 2.21)	1.01 (0.67 to 1.52)	1.45 (0.79 to 2.69)
2015-2016	NC	NC	NC	NC
**Time between cancer diagnosis and birth**				
Diagnosis <1 year	NC	NC	NC	NC
1 to <2 years	1.34 (0.82 to 2.21)	1.33 (0.77 to 2.29)	0.92 (0.58 to 1.46)	1.11 (0.56 to 2.19)
2 to <5 years	1.05 (0.83 to 1.34)	0.98 (0.73 to 1.31)	0.81 (0.62 to 1.06)	1.11 (0.79 to 1.56)
5 to <10 years	1.09 (0.81 to 1.47)	1.06 (0.74 to 1.51)	0.81 (0.58 to 1.12)	0.81 (0.48 to 1.38)
10 to <14 years	NC	NC	NC	NC
**Any chemotherapy**				
No	1.16 (0.95 to 1.42)	1.09 (0.86 to 1.38)	0.85 (0.68 to 1.06)	1.01 (0.74 to 1.37)
Yes	0.98 (0.71 to 1.36)	0.89 (0.61 to 1.32)	0.76 (0.55 to 1.06)	1.06 (0.66 to 1.72)
Any alkylating agent chemotherapy	0.96 (0.67 to 1.38)	0.95 (0.62 to 1.46)	0.76 (0.53 to 1.10)	1.07 (0.64 to 1.77)
Both alkylating and anthracycline chemotherapy	1.00 (0.67 to 1.47)	0.98 (0.61 to 1.56)	0.87 (0.58 to 1.29)	0.95 (0.54 to 1.68)

a Adjusted for year of birth, maternal age, race and ethnicity, marital status, maternal smoking during pregnancy. LBW = low birth weight; SGA = small for gestational age; NC = not calculated due to small number of outcomes.

## Discussion

Using health system data from California and population-based data sources and administrative claims from North Carolina, we compared the risk of adverse birth outcomes between births to AYA cancer survivors and matched births to women without a cancer history. Overall, we did not find a significant increase in the risk of preterm birth, LBW, SGA, low Apgar score, or any birth defect among births to cancer survivors. Stratified analyses also did not indicate an increased risk of these outcomes in subgroups defined by maternal and cancer characteristics, other than a suggested higher risk of preterm birth and/or LBW among births to gynecologic cancer survivors and cancer survivors who had an obese pre-pregnancy BMI or who smoked during pregnancy. Of note, chemotherapy exposure did not appear to be associated with increased risk of adverse birth outcomes in our cohort.

Several European studies, most including women diagnosed with cancer prior to the year 2000, have reported a significant increase in preterm births and LBW among cancer survivors compared to the general population, with relative risks generally ranging from about 1.2 to 2.0 for both outcomes (3-7,9,10). US-based studies are fewer in number but have often had similar findings. A recent study of commercially insured women from across the United States reported a slightly but significantly higher risk of preterm delivery among births to AYA cancer survivors (N = 2654) compared to births to women without cancer (RR = 1.19; 95% CI: 1.06 to 1.34) (15). Similarly, in our prior analyses using statewide birth certificate and cancer registry data in North Carolina, births to women diagnosed with AYA cancer before the start of pregnancy were 1.23 (95% CI: 1.07 to 1.43) and 1.36 (95% CI: 1.16 to 1.59) times as likely to be preterm and LBW, respectively, as births to cancer-free women (16). Although we did not observe a statistically significant difference in the risk of preterm or LBW infants between AYA cancer survivors and women without cancer in the current study, our overall estimates are not inconsistent with a small increase in risk of these outcomes after cancer. Given the totality of the evidence to date, continued surveillance of pregnancies in this population may still be warranted.

The risk of SGA was somewhat lower among births to AYA cancer survivors compared to births to women without a cancer history in our cohort. Most other studies have found no significant difference in SGA risk between births to women with and without cancer. In a recent meta-analysis of studies of women diagnosed with cancer before age 40 years, the risk of SGA was similar (RR = 0.99, 95% CI: 0.81 to 1.22) to that among women without cancer (2). As some studies have suggested (7,16), this finding may indicate that any observed increases in risk of LBW among survivors in previous reports are likely the result of an earlier delivery, rather than growth restriction in utero. Our results and those of others may therefore provide some reassurance regarding the risk of SGA infants among women with an AYA cancer history.

Our analyses stratified by cancer site suggested a potential increase in risk of both preterm birth and LBW among infants born to women diagnosed with gynecologic cancers as AYAs. Given the relatively small sample size of women with these cancers (N = 87), we did not conduct analyses for the more specific cancer types (cervical, ovarian, uterine) within this group, so these results should be interpreted with caution. An increased risk associated with gynecologic cancers is plausible, however, given the anatomic location of these malignancies and the treatments that women with these cancers may receive. Trachelectomy for cervical cancer, for example, involves surgical removal of the cervix, potentially leading to a greater risk of obstetric complications (26). Counseling on the potential adverse effects of cancer treatments on future birth outcomes may be especially critical for AYA women diagnosed with gynecologic cancers. For births to women with cancers other than gynecologic, we observed little difference in risk of adverse outcomes compared with matched births to women without cancer. These results are consistent with most prior studies for melanoma and thyroid cancer (16,27-29) but contrast with some findings for breast cancer and lymphomas. A meta-analysis of reproductive outcomes after breast cancer found increased odds of preterm birth (OR = 1.45; 95% CI: 1.11 to 1.88), LBW (OR = 1.50; 95% CI: 1.31 to 1.73), and SGA (OR = 1.16; 95% CI: 1.01 to 1.33) for breast cancer survivors compared to the general population (30). Some studies, although not all, have also reported elevated risks of these outcomes among women with hematologic cancers such as lymphoma (16,31,32). However, prior investigations have often included a high proportion of women diagnosed and treated before the year 2000, and therefore may not reflect current cancer treatment exposure patterns. Although we did not find an increased risk after AYA breast cancer or lymphoma diagnosed during 2003-2016, additional research, focused on AYAs treated with contemporary treatment protocols, may be needed to address the potential for excess risk of adverse outcomes in births to women with these cancer types.

It has been hypothesized that exposure to some chemotherapies could contribute to adverse birth outcomes through cardiovascular or pulmonary impairments (33,34), although few prior studies have examined these associations among AYAs (11,16). Notably, in the current study, neither exposure to any chemotherapy nor exposure to alkylating agents specifically was associated with a higher risk of preterm delivery or LBW among births to AYA cancer survivors compared to births to women without a history of cancer. Likewise, a recent study of women identified using the US-based Healthcare Cost and Utilization Project-Nationwide Inpatient Sample (HCUP-NIS) also did not find significant associations between prior chemotherapy exposure and preterm birth or intra-uterine growth restriction (35). Although our findings add to the evidence base on the potential adverse impact of chemotherapy history on obstetric outcomes, studies on these associations in the AYA cancer context remain limited.

Our stratified results suggested a higher risk of preterm birth and/or low birth weight among births to AYA women who had an obese pre-pregnancy BMI or who smoked during pregnancy. Survivors with these characteristics may represent additional groups to target for increased surveillance and counseling before delivery. Our findings were based on relatively small numbers of births, however, and further study of these associations is warranted.

It is notable that use of ART within 12 months of first post-diagnosis birth was uncommon among AYA cancer survivors in our study and similar to ART use among first included births to women without a cancer history. This suggests that higher risks of adverse birth outcomes among cancer survivors reported in previous studies may not be driven by differential rates of ART use between groups. Further investigation of relationships between cancer history, ART use, and birth outcomes would aid in the reproductive counseling of AYA patients.

Strengths of this study include the use of data sources that reflect contemporary cancer treatment strategies and childbearing patterns from 2 demographically diverse US states. Our use of administrative claims and integrated health care data allowed us to improve on the quality of cancer treatment information compared to our previous publications on this topic (16,36,37). Additionally, we were able to explore the potential contribution of assisted reproductive technology use to our findings. Our study also has some limitations. Although comparable to that of many other studies of reproductive outcomes after cancer, our statistical power was limited in some stratified analyses, and our sample size was insufficient to conduct extensive cancer-site-specific analyses of birth outcomes according to maternal characteristics or cancer treatments. Additionally, too few women in our cohort received pelvic radiation or total body irradiation for us to examine associations with these therapies. We also lacked individual-level information on some maternal characteristics, such as body mass index, comorbidities, and socioeconomic status, which could confound associations between cancer history and birth outcomes. However, we were able to account for factors such as maternal race and ethnicity, marital status, and smoking during pregnancy. Cancer recurrence information was also not available for our analyses, so we were unable to consider its impact on associations with adverse birth outcomes. Interpretation of our findings should also consider that all women included in our analyses were insured; it is unclear how inclusion of AYA women without health insurance would alter our results. However, recent US census data suggest that most individuals in the AYA age range (>85%) have health insurance (38). Births to women who disenrolled from KPNC or KPSC or moved out of North Carolina between cancer diagnosis and delivery would also not be included in our analyses. Based on census data for North Carolina from 2000-2010 (39), we estimate that approximately 15% of AYA women may have moved out-of-state during the study period, suggesting that we were able to identify most post-diagnosis births among women with AYA cancer in North Carolina. A prior analysis from KPSC found that retention rates in the health system after an AYA cancer diagnosis were 88%, 64%, and 52% at 1, 5, and 10 years, respectively (40). Retention was not associated with ethnicity but was lower among patients who were younger at diagnosis and higher among patients with breast cancer. Although we acknowledge the potential for missed births due to out-of-state moves or disenrollment, our stratified analyses did not suggest clear differences in associations according to factors that might predict moves and/or disenrollment, such as age or race and ethnicity. Additionally, we did not find a higher risk of adverse birth outcomes among AYA cancer survivors in North Carolina, where births were captured using statewide birth certificate records; therefore, continuous enrollment in insurance was not required for births to be observed. Taken together, these factors give us confidence in the validity of our findings.

In this study, we did not observe a significantly increased risk of adverse birth outcomes among AYA cancer survivors, other than those with gynecologic cancers. Our results also did not suggest a higher risk of these outcomes after chemotherapy exposure. Although these findings may provide some reassurance to women who want to have biological children after AYA cancer, they should be interpreted in the context of prior reports on these associations. Additional studies with cancer-site-specific analyses according to treatment and other characteristics would further our understanding of reproductive outcomes in this population.

## Supplementary Material

pkad106_Supplementary_Data

## Data Availability

The data underlying this article were provided to the authors under data use agreements. Due to privacy and ethical concerns, the data cannot be made available to the public.
