# Supplementary material for: Risk of adverse birth outcomes after adolescent and young adult cancer
Source: JNCI Cancer Spectr. 2023 Dec 21;8(1):pkad106. doi: 10.1093/jncics/pkad106 (PMC10868397; doi:10.1093/jncics/pkad106)
Supplement: pkad106_Supplementary_Data [file pkad106_supplementary_data.pdf]

Supplementary Materials

Supplementary Table 1. Maternal characteristics at first post-diagnosis births among births to AYA cancer survivors and matched births to women without cancer, stratified by study site

	North Carolina				KPSC				KPNC			
	Cancer (N=357)		No cancer (N=1718)		Cancer (N=397)		No cancer (N=1937)		Cancer (N=514)		No cancer (N=2411)	
	N	%	N	%	N	%	N	%	N	%	N	%
**Maternal age at birth**												
16-24	50	14%	240	14%	21	5%	105	5%	25	5%	110	5%
25-29	90	25%	435	25%	70	18%	345	18%	75	15%	343	14%
30-34	113	32%	547	32%	167	42%	814	42%	175	34%	823	34%
35-39	74	21%	355	21%	107	27%	523	27%	186	36%	880	36%
40-45	30	8%	141	8%	32	8%	150	8%	53	10%	255	11%
Median (IQR)	31	27-35	31	27-35	32	30-36	32	30-36	34	30-37	34	31-37
**Year of birth**												
2003-2006	30	8%	141	8%	20	5%	96	5%	9	2%	38	2%
2007-2010	131	37%	633	37%	85	21%	416	21%	127	25%	593	25%
2011-2013	124	35%	600	35%	100	25%	489	25%	106	21%	496	21%
2014-2019	72	20%	344	20%	192	48%	936	48%	272	53%	1284	53%
**Maternal race/ethnicity**												
Non-Hispanic white	270	76%	1020	59%	146	37%	551	28%	282	55%	962	40%
Non-Hispanic black	60	17%	315	18%	27	7%	137	7%	20	4%	189	8%
Hispanic	13	4%	271	16%	174	44%	953	49%	88	17%	586	24%
Other	14	4%	112	7%	50	13%	296	15%	124	24%	674	28%
**Marital status at birth**												
Not married	97	27%	521	30%	106	27%	467	24%	306	60%	1398	58%
Married	260	73%	1197	70%	291	73%	1470	76%	208	40%	1013	42%
**Tobacco use during pregnancy** [a]												
No	296	92%	1432	92%	373	98%	1800	98%	494	97%	2286	96%
Yes	27	8%	123	8%	8	2%	40	2%	13	3%	88	4%
Unknown/Missing	34		163		16		97		7		37	
**Pre-pregnancy body mass index** [b]												
Underweight (<18.5 kg/m2)	2	1%	33	4%	11	3%	26	1%	5	1%	33	2%
Normal (18.5-24.9 kg/m2)	103	53%	446	48%	140	38%	704	40%	183	42%	732	40%
Overweight (25.0-29.9 kg/m2)	37	19%	221	24%	102	27%	516	30%	137	31%	536	29%
Obese (≥30.0 kg/m2)	51	26%	222	24%	118	32%	503	29%	114	26%	518	28%
Unknown/missing	164		796		26		188		75		592	
**Gestational diabetes** [b]												

No	181	92%	881	93%	336	85%	1633	84%	468	91%	2188	91%
Yes	15	8%	63	7%	610	15%	304	16%	46	9%	223	9%
Unknown/missing	161		774		0		0		0		0	
**Oocyte or embryo cryopreservation (without transfer) within 90 days of cancer diagnosis**												
No	354	99%	N/A	N/A	397	100%	N/A	N/A	501	97%	N/A	N/A
Yes	3	1%	N/A	N/A	0	0%	N/A	N/A	13	3%	N/A	N/A
**Use of assisted reproductive technologies within 12 months of birth**												
No	352	99%	1704	99%	391	98%	1923	99%	500	97%	2329	97%
Yes	5	1%	14	1%	6	2%	14	1%	14	3%	82	3%

[a] Unavailable for births in 2010 in North Carolina

[b] Unavailable for births prior to 2011 in North Carolina
